# Racism in Medicine 101: A Simple and Engaging Approach to Challenging Topics in Resident Medical Education

**DOI:** 10.7759/cureus.105568

**Published:** 2026-03-20

**Authors:** Anthony J Mell, Monica Germain, Gopal Yadavalli, Jeffrey Schneider, Emily C Cleveland Manchanda

**Affiliations:** 1 Pediatrics and Child Health, Boston Medical Center, Boston, USA; 2 Nursing, Boston Medical Center, Boston, USA; 3 Infectious Diseases, Boston Medical Center, Boston, USA; 4 Emergency Medicine, Boston Medical Center, Boston, USA

**Keywords:** adult learning theory, anti-racism in medical education, curriculum development and evaluation, graduate medical education (gme), racism

## Abstract

Introduction

One strategy for combatting the effects of racism in healthcare is to provide education on racism’s manifestations. However, implementing effective training is challenging because of its complexity and resource requirements. Our objective in this study was to determine whether a new racism in medicine workshop, intentionally designed through the use of adult learning theories to be easy to deliver, community-centered, and thoughtful, could improve participant confidence in understanding racism in medicine in a manner applicable across multiple specialties.

Methods

We conducted a single-site survey study that ran from June 2023 to December 2023 at an academic medical center and included participants across six departments. There were 83 participants, 88% (73) of whom completed both pre- and post-session surveys. We developed and implemented a novel racism in medicine workshop that utilized existing educational materials and facilitators with limited teaching expertise. The primary outcome was participants’ self-reported confidence in several learning domains. A secondary outcome was participants’ satisfaction with the workshop.

Results

There was a significant improvement in participants’ confidence across all learning domains, with notable improvement in participants’ ability to identify examples of racial inequity mitigation (39.7% vs. 90.5%, p < 0.05) and understanding of race-conscious medicine (39.7% vs. 90.5%, p < 0.05). Most participants strongly agreed that they were satisfied with the workshop (65.8%) and that it was relevant to their training (75.3%).

Conclusions

These results suggest that the Racism in Medicine 101 workshop is effective, well received, and may be particularly useful in teaching real-world examples through community-centered discussion and the use of adult learning theories. We believe this success also suggests that our model, which utilized preexisting educational materials and facilitators with limited experience, is a viable solution to the challenges of educating medical trainees and faculty about racism in medicine, specifically avoiding the challenges of relying on highly experienced facilitators and one-size-fits-all solutions. This model could be studied further by including more participants, incorporating more institutions, and potentially linking this training to changes in behavior or clinical outcomes. Finally, we believe that this model could be applied to other challenging subjects, including topics such as transgender health, ableism, and the foster care system.

## Introduction

Racism profoundly contributes to health inequities that permeate the United States healthcare system. Between 1999 and 2011, Black Americans experienced over 1.63 million excess deaths and 80 million excess years of life lost compared to their White peers [1]. These disparities play out across all aspects of healthcare and affect individuals throughout their lifespans [2,3]. Racism among healthcare providers contributes directly to health inequities and can manifest as providers who dismiss patients’ symptoms, provide inadequate treatment, and exclude minoritized patients from healthcare decision-making processes [4,5].

Healthcare-based anti-racism interventions can help address health inequities. A recent review highlighted key strategies for successful interventions, including leadership buy-in, commitment of dedicated resources, long-term partnerships with racially minoritized groups, and implementation of staff education and training [6]. However, the literature lacks substantive guidance on effective models for anti-racism training for medical students, residents, fellows, and faculty. Such training should include both content related to medicine’s history of racism and the ways racism continues to impact healthcare today. Some ideas offered to educate clinicians about racism in medicine include avoiding a “one-size-fits-all” approach and acknowledging the challenges of time constraints when working with busy physicians. However, concrete examples of effective training strategies are more challenging to identify, and frequently, racism and its impact on healthcare are either not discussed or only superficially reviewed in the healthcare setting [4,5].

Designing effective education and training on racism in medicine is difficult for many reasons. First, this type of education requires addressing complicated concepts such as the complex interactions between healthcare systems and systems of racism and oppression [5,6], surfacing and confronting personal beliefs that may conflict with institutional policies [6], developing training material that makes clear the applicability to the participants’ day-to-day clinical work [5], and integrating trainings into demanding clinical schedules [4]. Furthermore, educators must carefully avoid endorsing flawed re-imaginings of racism, such as cultural racism and neoliberal interpretations [7].

A skilled and experienced facilitator is often cited as an important, if not critical, component of effective anti-racism training [6]. Facilitators leading discussions about complex topics, such as how racism affects healthcare, must be able to manage challenging and sometimes deeply personal discussions. As a result, anti-racism trainings tend to rely on a small number of content experts who are often under-resourced and over-tasked, making it difficult to scale this work. A webinar format offers a potential solution to this barrier; however, webinars may limit participant engagement and often fail to develop community through dialogue and shared experiences.

We aimed to develop an alternative approach to anti-racism training that reduced the cognitive load for both facilitators and participants while preserving the thoughtfulness and complexity the subject matter demands and allowing for the development of community through dialogue that promotes reflection, shared experience, and open discussion about racism. The primary outcome of this study was participants’ self-reported confidence in several learning domains, including their ability to understand the historical context of racism and White supremacy in the United States, understand what is meant by “race is a social construct,” understand the relationship between historical racist policies and modern racial inequities in health outcomes, differentiate between interpersonal, institutional, and systemic racism, describe examples of interventions medical institutions have made to mitigate racial inequities in health outcomes, articulate the difference between race-based and race-conscious medicine, feel that their own institution is committed to improving racial inequities in health, and feel empowered to learn more about how to advance health equity. A secondary outcome was participants’ satisfaction with the workshop.

## Materials and methods

Workshop design and implementation

In late 2022, we designed a “Racism in Medicine 101” workshop using three complementary conceptual frameworks to ensure alignment with our aims, incorporating adult learning theories in an intentionally multi-theory model [8]. Cognitive load theory [9] guided our approach to using accessible content that was intentionally designed to mitigate the chances of overwhelming facilitators or participants. We incorporated the theory of reflective practice [10] to enhance learning through individual and shared reflection. Finally, we used social capital and situativity theory [11,12] to develop a learning environment that strengthens community through building trust, shared values, and developing relationships.

The workshop was structured around a preexisting lecture, *Racism in Medicine: Historical Foundations and Strategies for Advancing Health Equity, *which is freely available and eligible for CME credit through the American Medical Association (permission to use was given by Dr. Emily Cleveland Manchanda) [13]. We developed an interactive learning model in which participants collectively watched the lecture, with intentionally interspersed, facilitated, structured group discussions. No prerequisite knowledge was required of the learners. To ensure meaningful discussion, we created a facilitator’s guide with reflection questions and discussion prompts for each segment (Appendix A). Workshops began with facilitator introductions and overview material, followed by a structured yet dynamic learning format. Participants viewed segments of the recorded lecture with predetermined breaks for facilitated group discussion of specific topics, including (1) definitions of key terms and description of race as a social construct; (2) privilege and the groundwater approach [14]; (3) interpersonal racism, colorblind racism [7], and microaggressions; and (4) institutional racism, race-based versus race-conscious medicine, and systemic racism [5]. Facilitators did not require any prerequisite knowledge except having previously participated in the session and reviewed the facilitator’s guide before the session. Of note, the facilitators in this session were participants in a health equity fellowship training program but began leading this session at the very start of their fellowship, when their health equity-specific knowledge was comparable to that of an average recent nurse or residency graduate.

We refined the workshop based on input from our research team, including the faculty member who developed the video lecture and is an expert in racism in medicine. The workshops were launched in June 2023 and offered to residency and fellowship programs, as well as department leadership, across Boston Medical Center. All tools used in this study were created by the team, except for the video lecture *Racism in Medicine: Historical Foundations and Strategies for Advancing Health Equity* [13].

Study design

The reach of the workshop was measured by counting the number of workshops given, departments included, and learners at each session. Effectiveness was evaluated with anonymous, paired pre- and post-session surveys using Likert scale responses to assess confidence in knowledge of racism in medicine before and after each workshop (Appendix B, Appendix C, Appendix D). The surveys were created using best practices based on recommendations from the American Association for Public Opinion Research for survey development and general best survey practices in medical education but were not validated against external tools [15,16]. We chose a survey design because our objective was specific, and a survey was sufficient to answer the questions of effectiveness and acceptability. Our survey sample included any medical student, resident, fellow, or faculty who joined the workshop; participation was entirely optional with no reward or consequence for participation or lack thereof. Evaluative questions were measured using a Likert scale, including a neutral choice, “Undecided,” and negative and positive choices with consistent language and logical order. With the exception of the Boston Medical Center-specific questions, the evaluative components of the pre- and post-session surveys were identical. Participant demographics were collected through the pre-session survey. The study was reviewed by the Institutional Review Board at Boston Medical Center and determined to be exempt.

Setting and participants

This survey study was conducted at a single academic medical center and included participants across six departments. Six departments agreed to host the workshop. When participants began the workshop, they were given the surveys with instructions to complete the pre-session survey before the workshop started and the post-session survey after the workshop was completed. The survey was presented as optional. Time was built into the workshop for participants to complete both surveys. There were 83 participants, 88% (73) of whom completed both pre- and post-session surveys. Participants were predominantly resident physicians, with smaller representation from medical students, fellows, and faculty. There was no age limit. Most participants held terminal degrees (MD or DO), but a few medical students still completing their training also participated. There were no exclusion criteria.

Statistical methods

To analyze changes in pre- and post-survey responses to each Likert scale question, paired one-sided Wilcoxon signed-rank tests were used, as the data were paired to each learner and not assumed to be normally distributed. Statistical analysis was performed in R (R Foundation for Statistical Computing, Vienna, Austria). Some participants offered descriptive evaluations of the workshop, which contextualized the quantitative results.

## Results

From June to December 2023, the Racism in Medicine 101 workshop was delivered seven times across six different departments (one department hosted the workshop twice for two separate groups of participants). On average, there were 12 participants per workshop. The length of the workshop varied from 90 to 105 minutes, allowing adequate time for discussion, reflection, and breaks. A total of 83 participants attended, 88% of whom completed both pre- and post-session surveys. Participant demographics (Table 1) reflect representation from multiple racial and ethnic groups: 48.4% Asian, 26.0% White, 16.4% Hispanic, Latino, or of Spanish origin, 6.85% Black, 5.48% Middle Eastern or North African, and 4.11% multiracial. Gender distribution showed that 61.6% identified as women, 37.0% as men, and 1.37% as other. The majority of participants were resident physicians (41.1% in postgraduate year 2 and 27.4% in postgraduate year 1), with the remainder including medical students (2.73%), fellows (2.74%), faculty (2.74%), and those in postgraduate years 3 and 4 or higher (13.7% and 9.59%, respectively).

**Table 1:** Demographics of participants in Racism in Medicine 101 workshops ^*^ Only showing categories with values ≥1

Characteristic	n	%
Race^*^		
Asian	28	48.4%
White	19	26.0%
Hispanic, Latino, or Spanish origin	12	16.4%
Black	5	6.85%
Middle Eastern or North African	4	5.48%
Multiracial	3	4.11%
Other	1	1.37%
Prefer not to answer	1	1.37%
Total	73	100%
Gender		
Cisgender man	27	37.0%
Cisgender woman	45	61.6%
Other	1	1.37%
Total	73	100%
Level of medical training		
Medical student	2	2.73%
Postgraduate year 1	20	27.4%
Postgraduate year 2	30	41.1%
Postgraduate year 3	10	13.7%
Postgraduate year 4 or higher	7	9.59%
Fellow	2	2.74%
Faculty	2	2.74%
Total	73	100%
Prior formal education on racism in medicine		
Yes	58	79.5%
No	15	20.5%
Total	73	100%

Analysis of the survey data showed a significant difference in median rank scores between the pre- and post-survey responses for each question, and the mode of Likert scale responses increased for five of the eight questions (Table 2). The Racism in Medicine 101 workshop increased participant agreement to every prompt provided, including their ability to understand the historical context of racism and White supremacy in the United States (74% vs. 89%, p < 0.05), understand what is meant by “race is a social construct” (74% vs. 95.9%, p < 0.05), understand the relationship between historical racist policies and modern racial inequities in health outcomes (75.3% vs. 91.7%, p < 0.05), differentiate between interpersonal, institutional, and systemic racism (78% vs. 94.5%, p < 0.05), describe examples of interventions medical institutions have made to mitigate racial inequities in health outcomes (39.7% vs. 90.5%, p < 0.05), articulate the difference between race-based and race-conscious medicine (39.7% vs. 90.5%, p < 0.05), feel Boston Medical Center is committed to improving racial inequities in health outcomes (67.1% vs. 82.2%, p < 0.05), and feel empowered to learn more about how to advance health equity (75.3% vs. 93.2%, p < 0.05).

**Table 2:** Pre- and post-session survey results from participants in the Racism in Medicine 101 workshops

Statement	Pre-session distribution				Post-session distribution				Statistical testing		
	Strongly disagree, disagree, and undecided		Agree and strongly agree		Strongly disagree, disagree, and undecided		Agree and strongly agree				
	%	n	%	n	%	n	%	n	p-value	Test statistic	Test performed
I understand the historical context of racism and White supremacy in the United States.	26.0%	19	74%	54	11.0%	8	89%	65	<0.05	V = 54	Paired one-sided Wilcoxon signed-rank test
I understand what is meant by “race is a social construct”.	26.0%	19	74.0%	54	4.1%	3	95.9%	70	<0.05	V = 0	Paired one-sided Wilcoxon signed-rank test
I understand the relationship between historical racist policies and modern racial inequities in health outcomes.	24.7%	18	75.3%	55	8.22%	6	91.7%	67	<0.05	V = 15	Paired one-sided Wilcoxon signed-rank test
I can differentiate between interpersonal, institutional, and systemic racism.	22%	16	78%	57	5.5%	4	94.5%	69	<0.05	V = 12	Paired one-sided Wilcoxon signed-rank test
I can describe examples of interventions medical institutions have made to mitigate racial inequities in health outcomes.	60.3%	44	39.7%	29	9.5%	7	90.5%	66	<0.05	V = 16.5	Paired one-sided Wilcoxon signed-rank test
I can articulate the difference between race-based and race-conscious medicine.	60.3%	44	39.7%	29	9.59%	7	90.5%	66	<0.05	V = 14	Paired one-sided Wilcoxon signed-rank test
I feel Boston Medical Center is committed to improving racial inequities in health outcomes.	32.9%	24	67.1%	49	17.8%	13	82.2%	60	<0.05	V = 56	Paired one-sided Wilcoxon signed-rank test
I feel empowered to learn more about how to advance health equity.	24.7%	18	75.3%	55	6.8%	5	93.2%	68	<0.05	V = 20	Paired one-sided Wilcoxon signed-rank test

The questions with the greatest change in responses included participants’ ability to describe examples of interventions medical institutions have made to mitigate racial inequities in health outcomes and to articulate the difference between race-based and race-conscious medicine. This suggests that the workshop may be particularly strong in helping ground the theoretical concepts of racism in medicine in real-world examples.

Qualitative evaluations suggested that the workshop’s effectiveness stemmed from the facilitators’ approach and the intentional framing of the discussion and content. One participant said, “I thought that the facilitator for this session was phenomenal. I appreciated the safe space and all of the great pearls. This session exceeded my expectations.” Another participant responded, “This is the first (and I’ve been involved in probably 20+ DEI, racism, etc. lectures) time I feel like I learned something. The lecture was good, but it was your (the facilitator’s) insight and moderation that were impactful.” These results highlight that participants had meaningful learning experiences attributed to the facilitators. Our findings suggest that facilitators with limited expertise can be highly effective when provided with sufficient support, in this case, a structured didactic format, a facilitator guide, and mentorship for leading these sessions.

Post-session survey questions on the acceptability of the material showed that a large majority of participants strongly agreed that they were satisfied with the quality of the workshop (65.8%) and that the session was relevant to their medical training (75.3%) (Table 3). This suggests that the workshop was widely acceptable and well received by learners across a range of departments and a diverse set of learners in regard to race, gender, and level of training.

**Table 3:** Post-session survey results on satisfaction and relevance of the Racism in Medicine 101 workshop

Statement	Post-session distribution									
	Strongly disagree		Disagree		Undecided		Agree		Strongly agree	
	%	n	%	n	%	n	%	n	%	n
I was satisfied with the quality of this lecture and discussion.	0%	0	0%	0	4.11%	3	30.1%	22	65.8%	48
This lecture was relevant to my medical training.	0%	0	0%	0	4.11%	3	20.5%	15	75.3%	55

## Discussion

Our results demonstrate that the workshop was effective and well received and that it may be particularly effective in grounding theoretical concepts in real-world examples. Consequently, this workshop offers a solution to the challenges of providing effective and meaningful education on racism in medicine across a diverse set of learners in an academic medical center, while requiring minimal expertise and preparation and utilizing preexisting materials. Furthermore, the acceptability of this model suggests that, despite using conserved materials across departments, the addition of facilitated, community-centered discussions may address the “one-size-fits-all” challenge without being overly burdensome.

The success of the workshop was grounded in its design and the integration of key principles of the selected conceptual frameworks. We applied cognitive load theory by utilizing peer facilitators, specifically a junior faculty physician and a nurse enrolled in a health equity fellowship program. While these facilitators had some content knowledge, they were still developing experience and expertise in discussing racism in medicine. The format of combining a pre-recorded lecture with guided discussions led by near-peer facilitators with minimal expertise created meaningful discussions. To reduce the cognitive load for physician participants, we eliminated pre-work, provided focused learning objectives with a narrow scope, and alternated between passive video learning and active engagement in meaningful discussions. We applied reflective practice theory by dedicating time for in-person reflection and discussions that encouraged collective learning. Additionally, we applied social capital theory by conducting workshops in established academic department spaces, enabling participants to engage with the material as a community and incorporate the learning into existing departmental relationships, didactic spaces (e.g., routine conference time), and culture.

Some limitations of this work include the fact that the survey was promoted by department leadership, who requested to host the workshops. The framing of the workshop as a department-leader priority could have influenced participants’ views of the workshop; however, we would expect that effect to be strongest before the workshop and to have less impact on the comparison of pre- and post-session responses. Another limitation is that no non-responder analysis was completed. It is possible that participants dissatisfied with the workshop chose not to complete the survey. However, given the very high survey response rate of 88%, we are reassured about the quality of our results. Similarly, the use of a non-validated survey weakens our findings. At the time of the study, however, no validated surveys in this subject area were available.

## Conclusions

To further test the effectiveness and generalizability of the Racism in Medicine 101 workshop, we plan to implement it in additional departments at Boston Medical Center and at other healthcare institutions. Our innovative content delivery model, which facilitates community-based discussions of challenging topics using publicly available materials and minimally trained facilitators, has potential applications beyond racism in medicine. This approach could be adapted to address other challenging subjects in medicine, such as transgender health, addressing ableism in medical education, and the foster care system. The model’s key strength is its ability to create meaningful community dialogue and facilitate cultural change while minimizing demands on hospital or residency leadership. By demonstrating that effective facilitation does not require subject matter expertise, our workshop model offers a sustainable and scalable approach to addressing sensitive topics in medical education. Additionally, it would be beneficial to study the impacts of this workshop or similar models not only on participant confidence but also on behavior or clinical outcomes, thereby linking education to real-world impact.

The results of this study show that the Racism in Medicine 101 workshop was effective and well received. The workshop may also be particularly effective in grounding theoretical concepts in real-world examples. This model could be scaled across multiple institutions and applied to other challenging subjects in medicine that present similar educational challenges.

## Figures and Tables

**Figure 1:**
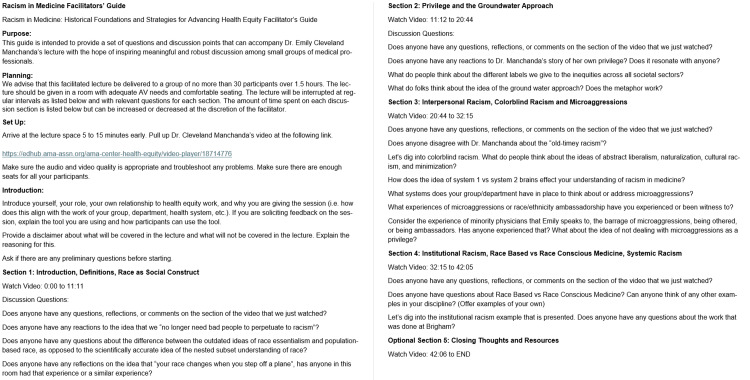
Racism in Medicine facilitator’s guide

## Appendices

Appendix A

Appendix B 

**Table 4:** Pre-session demographic questions

Questions	Answer options
What is your race/racial identity? Please select all that apply.	Asian
	Native Hawaiian or Other Pacific Islander
	Black or African American
	Hispanic, Latino, or Spanish Origin
	Middle Eastern or North African
	Native American or Alaskan Native
	White or Caucasian
	Multiracial or Biracial
	Other
	Prefer not to answer
What is your gender/gender identity? Please select all that apply.	Cisgender Man
	Cisgender Woman
	Transgender Man
	Transgender Woman
	Non-binary
	Other
	Prefer not to answer
What is your current level of medical training?	Medical Student
	Resident PGY1
	Resident PGY2
	Resident PGY3
	Resident PGY4 or greater
	Fellow PGY1
	Fellow PGY2
	Fellow PGY3
	Fellow PGY4 or greater
	Faculty Physician
	Prefer not to answer
Have you ever previously received formal education around racism in medicine (e.g., a lecture, course, interactive workshop, or other training)?	Yes
	No

Appendix C  

**Table 5:** Pre-session assessment tool Instructions: Please indicate your level of agreement with each of the following statements (scale is 1 Strongly disagree, 2 Disagree, 3 Undecided, 4 Agree, and 5 Strongly agree).

Questions	Answer options
I understand the historical context of racism and white supremacy in the United States.	1
	2
	3
	4
	5
I understand what is meant by “race is a social construct”.	1
	2
	3
	4
	5
I understand the relationship between historical racist policies and modern racial inequities in health outcomes.	1
	2
	3
	4
	5
I can differentiate between interpersonal, institutional, and systemic racism.	1
	2
	3
	4
	5
I can describe examples of interventions medical institutions have made to mitigate racial inequities in health outcomes.	1
	2
	3
	4
	5
I can articulate the difference between race-based and race-conscious medicine.	1
	2
	3
	4
	5
I feel BMC is committed to improving racial inequities in health outcomes.	1
	2
	3
	4
	5
I feel empowered to learn more about how to advance health equity.	1
	2
	3
	4
	5

Appendix D

**Table 6:** Post-session assessment tool Instructions: Please indicate your level of agreement with each of the following statements (scale is 1 Strongly disagree, 2 Disagree, 3 Undecided, 4 Agree, and 5 Strongly agree).

Questions	Answer options
I understand the historical context of racism and white supremacy in the United States.	1
	2
	3
	4
	5
I understand what is meant by “race is a social construct”.	1
	2
	3
	4
	5
I understand the relationship between historical racist policies and modern racial inequities in health outcomes.	1
	2
	3
	4
	5
I can differentiate between interpersonal, institutional, and systemic racism.	1
	2
	3
	4
	5
I can describe examples of interventions medical institutions have made to mitigate racial inequities in health outcomes.	1
	2
	3
	4
	5
I can articulate the difference between race-based and race-conscious medicine.	1
	2
	3
	4
	5
I feel BMC is committed to improving racial inequities in health outcomes.	1
	2
	3
	4
	5
I feel empowered to learn more about how to advance health equity.	1
	2
	3
	4
	5
This lecture was relevant to my medical training	1
	2
	3
	4
	5
I feel empowered to learn more about how to advance health equity	1
	2
	3
	4
	5
